# Nanoparticles for Augmenting Therapeutic Potential and Alleviating the Effect of Di(2-ethylhexyl) Phthalate on Gastric Cancer

**DOI:** 10.1021/acsami.3c15976

**Published:** 2024-04-04

**Authors:** Hau-Lun Huang, Kuo-Wei Chen, Hsiao-Wei Liao, Ling-Yu Wang, Shin-Lei Peng, Chih-Ho Lai, Yu-Hsin Lin

**Affiliations:** † Department of Pharmacy, National Yang Ming Chiao Tung University, Taipei 112304, Taiwan; ‡ Division of Hematology and Oncology, Cheng Hsin General Hospital, Taipei 112401, Taiwan; § Institute of Pharmacology, National Yang Ming Chiao Tung University, Taipei 112304, Taiwan; ∥ Department of Biomedical Imaging and Radiological Science, China Medical University, Taichung 40402, Taiwan; ⊥ Department of Microbiology and Immunology, Molecular Infectious Disease Research Center, Chang Gung University and Chang Gung Memorial Hospital, Taoyuan 33302, Taiwan; # Medical Device Innovation and Translation Center, National Yang Ming Chiao Tung University, Taipei 112304, Taiwan; ∇ Department of Medical Research, China Medical University Hospital, China Medical University, Taichung 40402, Taiwan

**Keywords:** di(2-ethylhexyl) phthalate, gastric cancer, epithelial-mesenchymal transition, nanoparticles, TPGS-conjugated fucoidan

## Abstract

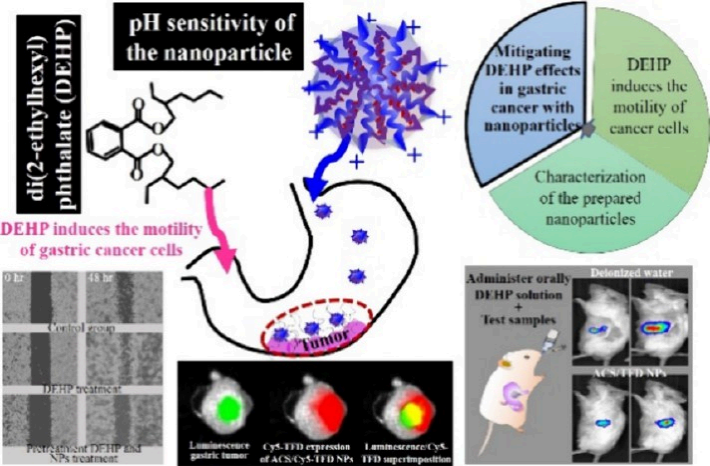

Changes in diet culture and modern lifestyle contributed to a higher incidence of gastrointestinal-related diseases, including gastritis, implicated in the pathogenesis of gastric cancer. This observation raised concerns regarding exposure to di(2-ethylhexyl) phthalate (DEHP), which is linked to adverse health effects, including reproductive and developmental problems, inflammatory response, and invasive adenocarcinoma. Research on the direct link between DEHP and gastric cancer is ongoing, and further studies are required to establish a conclusive association. In our study, extremely low concentrations of DEHP exerted significant effects on cell migration by promoting the epithelial-mesenchymal transition in gastric cancer cells. This effect was mediated by the modulation of the PI3K/AKT/mTOR and Smad2 signaling pathways. To address the DEHP challenges, our initial design of TPGS-conjugated fucoidan, delivered via pH-responsive nanoparticles, successfully demonstrated binding to the P-selectin protein. This achievement has not only enhanced the antigastric tumor efficacy but has also led to a significant reduction in the expression of malignant proteins associated with the condition. These findings underscore the promising clinical therapeutic potential of our approach.

## 1 Introduction

Changes in modern diet culture and lifestyle have led to an increase in the prevalence of gastrointestinal-related diseases, particularly long-term or severe digestive system diseases that could increase the risk of gastric cancer.^1−3^ Studies have demonstrated a significant association between consuming processed foods rich in salt, pickled foods, and processed meat and an increased risk of gastric cancer, which is particularly prevalent in Asian countries.^4^ Furthermore, some Asians face increased exposure to plasticizers, such as phthalates and bisphenols, compared to other populations, primarily due to the widespread utilization of plastic products.^5,6^ Di(2-ethylhexyl) phthalate (DEHP), the predominant plasticizer used to make polyvinyl chloride (PVC) products flexible, such as medical devices and pharmaceuticals, contributes up to 40% of the weight of intravenous bags and is present in more than 80% of the weight of medical tubing.^7,8^ DEHP is also used to manufacture flexible plastics for various applications, such as food packaging, indoor decorations, and children’s toys. These plastics pose an exposure risk with contact by chemical leaching into the packaging material.^2^ This raises concerns about exposure to DEHP, which has been associated with adverse health effects, such as reproductive and developmental problems and an inflammatory response. DEHP has the potential to exacerbate poly formation and invasive adenocarcinoma in affected individuals.^9−11^ Given the ongoing research aimed at establishing an association between phthalate exposure and gastric cancer, we were particularly interested in investigating the potential link between the plasticizer DEHP and the progression of gastric cancer.

**Figure 1:**
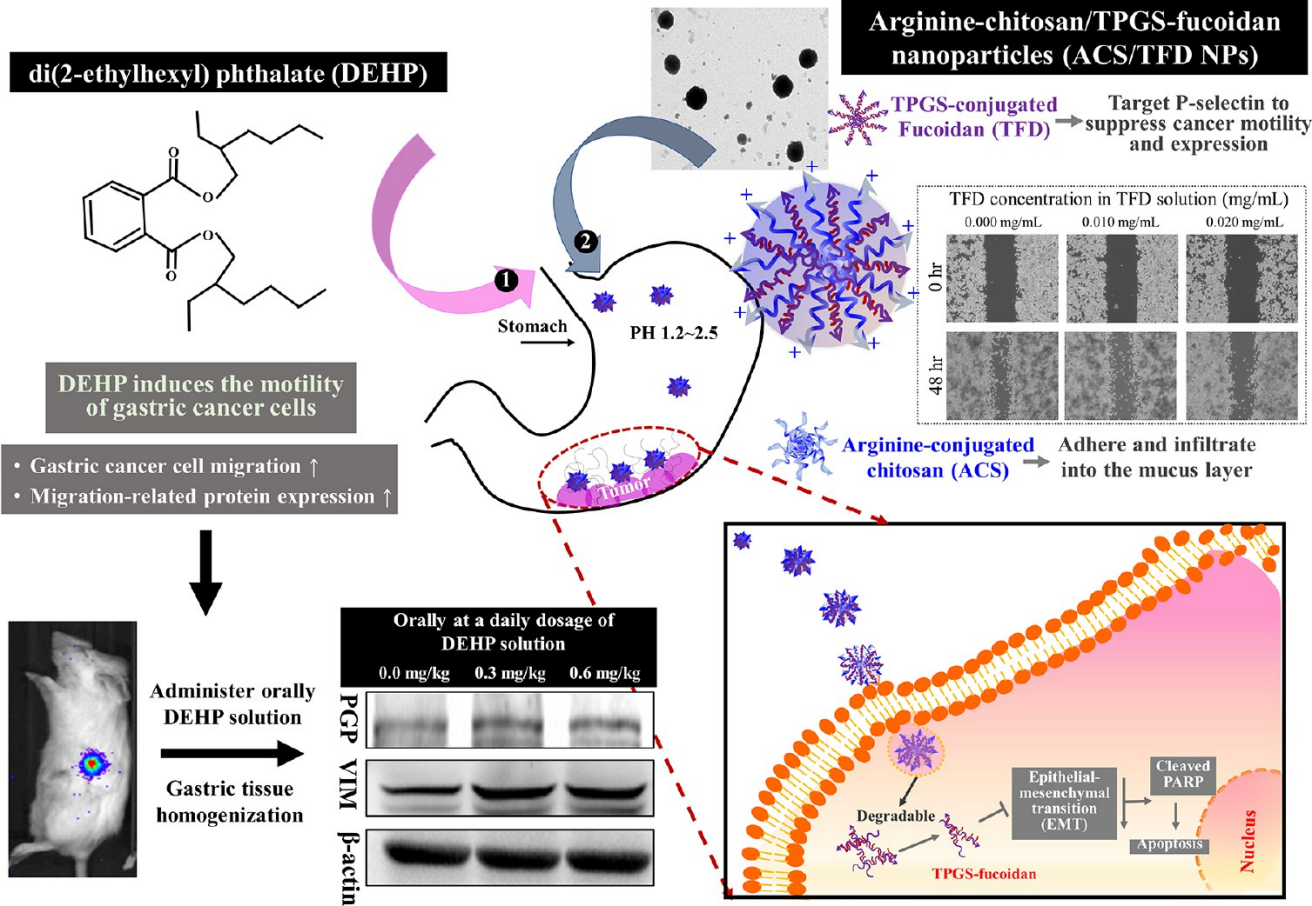
Schematic diagram of illustrating the prepared ACS/TFD NPs, designed to enhance therapeutic efficacy while mitigating the impact of DEHP on gastric cancer therapy.

Chemotherapy is the first-line and most effective modality for treating cancers when used alone or in combination with other treatments, such as surgery and radiation therapy.^12^ A characteristic of chemotherapeutic drugs is their inability to differentiate between cancer cells and normal cells, resulting in toxicity and adverse effects. Long-term chemotherapy treatment may lead to drug resistance in tumor cells, reducing the treatment’s effectiveness.^13−15^ Therefore, research and development of new drugs and effective treatments for cancer therapy is important. d-α-Tocopherol polyethylene glycol succinate (TPGS), or vitamin E TPGS, is a synthetic derivative of natural α-tocopherol that has received attention in drug delivery systems.^16^ TPGS has demonstrated the ability to inhibit growth of cancer cells, act as an antioxidant, and inhibit the activity of multidrug resistance proteins.^17^ Our initial development of TPGS-conjugated fucoidan (FD) demonstrated suppressing the migration of gastric cancer cells, resulting in a significant reduction in scratch wound coverage from 40.16 ± 3.15 to 26.96 ± 4.57% across concentrations ranging from 0.000 to 0.020 mg/mL (Figure 1). FD is a sulfated polysaccharide primarily derived from brown seaweed that has promising anticancer effects by selectively binding the transmembrane P-selectin protein and modulating various cellular processes involved in tumor growth and metastasis.^18^

Research in the past decade has been devoted to designing a nanoparticle (NP) delivery system to improve the stability of therapeutic agents against degradation in gastric acid and enable the interaction between the delivered drug and the targeted cancerous tissue after oral drug delivery.^19−21^

Arginine (ARG) is a basic amino acid with a positively charged guanidinium group that interacts with negatively charged surfaces, such as the mucus layer, to enhance mucoadhesion and cellular uptake.^22^ Orally administered ARG-conjugated polymers can improve drug absorption, stability, and bioavailability. Chitosan (CS) is a safe, polycationic, mucoadhesive, and biodegradable polymer that interacts with anionic moieties at the cell surface. It enables prolonged interactions between the delivered drug and membrane epithelia, thereby enhancing drug diffusion into the mucus/epithelial layer.^23,24^ CS is primarily transported through endocytosis, which involves two major pathways: phagocytosis and pinocytosis. Pinocytosis-mediated CS uptake can occur via caveolin, cadherin, and clathrin processes.^25^ In our study, we prepared positively charged NPs consisting of ARG-conjugated CS (ACS) and TPGS-conjugated fucoidan (FD) (TFD) for gastric cancer treatment. These NPs have the potential to enhance gastric residence time, facilitate mucus layer infiltration, and enable controlled release, thereby improving the oral delivery of gastric carcinoma therapies. Additionally, pH-sensitive NPs induce the disintegration and release of TFD, enabling an investigation into its role and interaction with P-selectin in DEHP-treated gastric cancer cells. Furthermore, *in vivo* distribution, antitumor efficacy, and safety were evaluated by assessing NP activity in a mouse model with orthotopic gastric tumors that were pretreated with a DEHP solution (Figure 1).

## 2 Experimental Section

### 2.1 Cell Culture and the Effects of DEHP on Cell Viability and Wound Healing

Human gastric cancer MKN45 cells were purchased from the Japanese Collection of Research Bioresources Cell Bank and were grown in an RPMI 1640 medium supplemented with 10% fetal bovine serum, 100 μg/mL streptomycin, and 100 U/mL penicillin (Hyclone, Logan, UT, USA). The MKN45 cells were seeded at densities of 1.0 × 10^4^ cells per well in 96-well plates and allowed to adhere overnight. The growth medium was then replaced with a medium containing varying DEHP concentrations for cell cytotoxicity assays. The cells were incubated for 24 to 48 h, and cell growth was assessed using the 3-(4,5-dimethylthiazol-2-yl)-2,5-diphenyltetrazolium bromide (MTT) assay. Moreover, the 1.0 × 10^5^ cell suspension was seeded into each well with the silicone culture-insert 2 well to perform the wound healing assay. The cells were incubated overnight at 37 °C to facilitate cell adhesion and spreading on the substrate. The inserts were then delicately removed by peeling them back from one corner as per the manufacturer’s instructions. Subsequently, a fresh culture medium containing the DEHP solution was added for coincubation. Finally, images were taken with an optical microscope (Olympus CKX53, Japan) at a magnification of 10× phase objective. The cell motility was calculated using the following equation:^26^
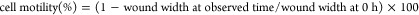


### 2.2 Analysis of Malignant-Related Protein Expression in DEHP-Treated MKN45 Cells

To study the influence of DEHP on the expression of proteins associated with malignancy in MKN45 cells, cells were treated with different concentrations of DEHP solution for 24 and 48 h. Whole-cell lysates from MKN45 cells were collected using a radioimmunoprecipitation assay (RIPA) buffer containing phosphatase inhibitors, and protein levels were quantified with the Bradford protein assay. Equal protein amounts were separated via 10% sodium dodecyl sulfate-polyacrylamide gel electrophoresis and transferred onto poly(vinylidene difluoride) (PVDF) membranes. The membranes were then blocked with defatted dry milk in phosphate-buffered saline (PBS) for a duration of 1 h. The proteins were detected by incubating the membranes with primary antibodies, including anti-P-glycoprotein (PGP), antivimentin (VIM), and anti-β-actin (β*-*actin), overnight at 4 °C. Finally, the membrane was exposed to horseradish peroxidase secondary antibody conjugates for 1 h and visualized using enhanced chemiluminescence (ECL) with a MultiGel-21-C2 imaging system (Topbio, Taiwan). The intensity of the bands was quantified using ImageJ software by measuring their optical density. Moreover, to observe these proteins' distribution in MKN45 cells after DEHP treatment, the cells were then incubated with primary antibodies against PGP or VIM followed by incubation with secondary antibodies conjugated with CF 594 for 1 h in the dark. The nuclei were counterstained with 4′,6-diamidino-2-phenylindole (DAPI) and carefully mounted on glass slides. Confocal laser scanning microscopy (CLSM) was used to visualize the fluorescence images, and the fluorescence intensity was quantified using MetaMorph software.^27^

### 2.3 Preparation and Characterization of ACS/TFD NPs

ACS was synthesized by reacting the carboxylic group of ARG with the primary amine group of CS using the coupling agents *N*-hydroxy-succinimide (NHS) and 1-ethyl-3-(3-(dimethylamino)propyl) carbodiimide (EDC), using a previously established method with some modifications.^28,29^ Initially, 2.0 g of ARG was dissolved in 50.0 mL of deionized water, and NHS and EDC were added to activate ARG’s carboxyl group. After the pH was adjusted to 6, the activation reaction proceeded for 2 h. Subsequently, CS (0.5 g) was dissolved in 0.5% acetic acid (50.0 mL), and the pH was adjusted to 6. The activated ARG solution was then added to the CS solution and incubated at ambient temperature with continuous stirring for 72 h to complete the reaction. Furthermore, the TFD copolymer was synthesized by grafting TPGS onto the carboxyl group of FD via esterification.^30,31^ To synthesize TFD, 0.8 g of FD was dissolved in 10 mL of deionized water, and 0.4 g of TPGS (average molecular weight: 1513 Da), dissolved in 5.0 mL of deionized water, was then added to the aqueous FD solution with continuous stirring at room temperature. Subsequently, dicyclohexylcarbodiimide (0.15 mmol) and 4-dimethylaminopyridine (0.15 mmol) were dissolved in 6 mL of acetonitrile, and 1 mL of triethanolamine was added to the mixture of aqueous TPGS/FD and stirred for 24 h to complete the reaction. The resultant ACS and TFD samples were dialyzed using a Spectra/Por membrane (molecular weight cutoff of 6000–8000 Da), with daily water replacement for 5 days to remove any unconjugated material. The quantification of the TPGS content in TFD was conducted using liquid chromatography–mass spectrometry (LC–MS), offering a reliable analytical approach for evaluating the TFD polymer (see the Supporting Information for detailed procedures). The purified ACS and TFD copolymers were collected after freeze-drying, and their quality was confirmed through analysis using Fourier transform infrared (FTIR) spectra and nuclear magnetic resonance (NMR). In order to determine the optimal preparation conditions for the ACS/TFD NPs, we examined NPs with varying ACS concentrations while maintaining a fixed TFD concentration. The NPs were synthesized using a dropwise method, where an aqueous solution of ACS (1.25, 2.50, 5.00, 7.50, and 10.00 mg/mL; 0.1 mL) was added drop by drop into an aqueous solution of TFD (0.625 mg/mL; 0.4 mL). The resulting mixture was gently shaken for 0.5 h at a temperature of 37 °C. Subsequently, the mixture was subjected to centrifugation, and a Zetasizer instrument was employed to determine the particle size, polydispersity index, and zeta potential of the samples.

### 2.4 pH Sensitivity of the ACS/TFD NP Morphology and Release Profiles

The pH-dependent behavior of the NPs was investigated by analyzing their morphological alterations using transmission electron microscopy (TEM) in various pH environments. The NPs were examined in pH 1.2 (simulating the gastric fluid with hydrochloric acid and pepsin buffer), pH 5.5 (sodium acetate buffer), pH 6.8, and pH 7.4 (phosphate-buffered saline; PBS), representing simulated environments of the gastric mucosa, extracellular tumor tissue, and intracellular tumor tissue, respectively.^32,33^ The NP suspension corresponding to each pH value was deposited onto a copper grid with a mesh structure and subsequently stained with osmium tetroxide to enhance morphology visualization. Meanwhile, to monitor the release of TFD from the NPs, the fluoresceinamine (FA)-labeled TFD (FA-TFD) was synthesized using a modified method, which was then followed by the production of fluorescent ACS/FA-TFD NPs using the previously described procedure.^34^ The release profiles of FA-TFD were studied in a simulated dissolution medium with varying pH levels, maintaining a constant temperature of 37 °C to mimic physiological conditions. At specific time intervals, samples were collected and centrifuged, and the resulting supernatants were analyzed with a microplate spectrofluorometer. The released amount of FA-TFD was quantified by employing a standard calibration curve. The release experiments were repeated five times for each condition.

### 2.5 The Effect of TFD in TFD Solution or ACS/TFD NPs on Cell Motility and Viability

Cells were subjected to treatment with either TFD solution or ACS/TFD NPs, and their motility was assessed using appropriate assays, such as wound healing assays. First, MKN45 cells were seeded at a density of 8.0 × 10^5^ cells in a 6 cm Petri dish and cultured for 1 day. Subsequently, the growth medium was replaced with a DEHP-containing medium, and the cells were incubated continuously for another 48 h. Cancer cells were seeded at a density of 1.0 × 10^4^ cells/well in 96-well plates and allowed to adhere overnight. On the following day, the cells were exposed to TFD solution or TFD/ACS NPs at different TFD concentrations for 24 h. Subsequently, cytotoxicity was evaluated using the MTT assay. In addition, the DEHP-treated cells at 1.0 × 10^5^ cells/well were seeded into the silicone culture-insert wells and incubated overnight to allow the cells to spread on the substrate. After carefully removing the culture-inserts, a fresh culture medium containing TFD solution or TFD/ACS NP solution was added followed by coincubation for 24 and 48 h. Finally, images were captured by using an optical microscope to analyze the effects of the treatment on cell motility.

### 2.6 Evaluation of Cellular Distributions and Immunofluorescence Staining Contained within Fluorescence NPs Using Confocal Microscopy

To observe the interaction of the TFD solution or ACS/TFD NPs on cell surface protein expression, the fluorescent fluorescein isothiocyanate (FITC)–ACS/cyanine 3 hydrazide (Cy3)–TFD NPs were produced following the procedure. In brief, the fluorescent dye-labeled polymer FITC–ACS synthesis involved adding FITC (5.0 mg/5.0 mL in dehydrated methanol) to an aqueous solution of ACS (50.0 mg/5.0 mL), enabling the reaction between the isothiocyanate group of FITC and the primary amino group of ACS.^35,36^ Moreover, the fluorescent polymer Cy3–TFD was synthesized by gently adding fluorescent solution [Cy3; 1.0 mg/0.1 mL in dimethyl sulfoxide (DMSO)] to an aqueous solution of TFD (0.2 g/20.0 mL). Fluorescent hydrazides, such as Cy3 and Cy5 hydrazides, function as carbonyl-reactive dyes, exhibiting a well-established capacity similar to that of fluorescein hydrazide, enabling the labeling of diverse carbonyl-containing molecules, such as proteins, antibodies, and polymers.^37−39^ Following the synthesis, the resultant mixtures were stirred in the dark at ambient conditions for 12 h, and then, the FITC–ACS or Cy3–TFD solution was dialyzed against 5 L of deionized water to remove any unbound fluorescent dye. Finally, the resulting fluorescence polymers were lyophilized using a freeze-dryer. Afterward, DEHP-treated cells at a concentration of 4.0 × 10^5^ cells/mL were cultured on glass coverslips and incubated at 37 °C for 24 h. The cells were exposed to the prepared fluorescent Cy3–TFD solution or FITC–ACS/Cy3–TFD NPs for 2 h. The cells were fixed by using a 3.7% paraformaldehyde solution and stained with DAPI to visualize the nuclei. CLSM was utilized to observe the stained cells, employing excitation wavelengths of 340, 488, and 525 nm. The binding capability of TFD to P-selectin was assessed by exposing the cells to either fluorescent Cy3–TFD solution or FITC–ACS/Cy3–TFD NPs for 24 h. After the treatment, the cells were subjected to overnight incubation at 4 °C with a rabbit anti-P-selectin primary antibody followed by a 1 h incubation in the dark with an antirabbit CF 633 secondary antibody. Afterward, the nuclei were stained with DAPI and examined using CLSM. The fluorescence images were quantitatively analyzed using MetaMorph software. To evaluate the binding specificity of TFD with P-selectin, human recombinant P-selectin was added to highly hydrophobic 96-well plates and incubated overnight at 4 °C. After a PBS wash, varying concentrations of Cy3–TFD solution were added to the wells for 1 h followed by three PBS washes. Simultaneously, P-selectin-coated wells were exposed to test fluorescent samples and an anti-P-selectin antibody for comparing binding specificity. The fluorescence intensity was measured using a microplate spectrofluorometer (see the Supporting Information for detailed procedures).

### 2.7 Analysis of the Interplay between DEHP and ACS/TFD NPs on the Expression of Metastasis-Related Proteins

To investigate the impact of DEHP on metastasis-related protein expression in MKN45 cells, the cells were treated with varying concentrations of DEHP for a duration of 48 h. After the treatment period, whole-cell lysates of DEHP-treated cells were collected and lysed by using a microwestern array (MWA) lysis buffer. Subsequently, an MWA technique utilizing specific antibodies was employed to detect the expression of various target protein expressions, with α-tubulin and β-actin serving as loading controls during the analysis. Protein bands were captured using an Odyssey infrared imaging system, and the corresponding intensities of the expressed proteins’ bands were quantified using Image Studio software (version 5.2; LI-COR Biosciences, USA).

To assess the effect of ACS/TFD NPs on the expression of metastasis-associated proteins in DEHP-treated MKN45 cells by conventional Western blotting, MKN45 cells were incubated with a DEHP-containing medium for 48 h followed by washing with DPBS, and the cells were treated with ACS/TFD NPs (containing TFD concentrations of 0.000, 0.020, and 0.040 mg/mL) for another 24 h treatment. On the following day, the cells were achieved using a lysis buffer supplemented with dithiothreitol, a combination of phosphatase inhibitors, and protease inhibitors. Then, antibodies against phosphor-PI3K (p-PI3K), PTEN, p-AKT, N-cadherin (NCAD), and VIM were obtained from Genetex (Irvine, CA, USA), while antibodies against p-PDK1, phospho-mTOR (p-mTOR), and E-cadherin (ECAD) were purchased from Cell Signaling Technology (Danvers, MA, USA). Finally, internal control antibodies, including β-actin and GAPDH antibodies, were obtained from Novus Biologicals (Littleton, CO, USA) and utilized to ensure an equal loading of samples. The signals on immunoreactive blots were subsequently visualized by employing ECL immunoblotting substrates.

### 2.8 Evaluation of Antitumor Activity through Tumor Bioluminescence, Immunohistochemistry, and Analysis of NP Distribution within the Tumor

Animal care and experimental procedures followed the 1996 edition of the Guide for the Care and Use of Laboratory Animals by the National Research Council’s Institute of Laboratory Animal Resources, published by the National Academy Press. Institutional Animal Care and Use Committee (IACUC 1100312) approval was obtained for all care guidelines and experimental protocols. Male six-weeks-old severe combined immunodeficiency (SCID) mice were utilized to establish the orthotopic gastric tumor model following our previously described procedure.^40^ After the stability of the bioluminescent gastric tumor was achieved, the experimental protocol involved initiating oral administration of a DEHP solution and administering various test sample treatments. The tumor growth was examined with an IVIS Luminar II *in vivo* imaging system (PerkinElmer, Waltham, MA, USA) by capturing bioluminescence tumor signals. Each group consisted of six mice and received different TFD formulations at a fixed dose of 50.0 mg/kg TFD, administered as a 0.5 mL volume. The TFD formulations included a TFD solution or ACS/TFD NPs. Simultaneously, the control group received either a 50.0 mg/kg ACS solution or a normal saline solution. These formulations were administered once daily for 15 consecutive days. Intraperitoneal injection of luciferin was administered to the mice followed by a 10 min waiting period to observe for bioluminescent expression. Images were captured by using a highly sensitive CCD camera and viewed in real time on a computer screen. The images were displayed using a color denoting the total flux in photons per second per square centimeter per steradian. The biosafety of nanosystems intended for clinical use is of significant concern.^41^ Healthy mice were divided into two groups: one received a normal saline solution, and the other was orally administered with ACS/TFD NPs once daily for 30 consecutive days. Throughout repeated dose studies, we closely monitored the body weights of all mice. Following the final observation, the animals were euthanized, and their organs were harvested and stained with hematoxylin and eosin for histological examination. The efficacy of ACS/TFD NPs in relation to tumor status was evaluated through immunohistochemical staining of VIM or NCAD, which are mesenchymal markers associated with the epithelial-mesenchymal transition (EMT), as well as cleaved PARP, an apoptotic marker. Subsequently, tissue inflammation and related protein expression were examined using a light microscope at different magnifications. Meanwhile, in the *in vivo* study on NP distribution, fluorescent-labeled ACS/cyanine 5 hydrazide (Cy5)–TFD NPs were administered orally through the esophagus using an oral feeding needle. After the mice were euthanized at various time points following treatment, fluorescence images of organs and tissues were acquired using an *in vivo* optical imaging system (Photon Imager Optima, Biospace Lab, France). Subsequently, immunofluorescence staining of gastric tumor slides was performed using a rabbit anti-P-selectin primary antibody followed by probing with a secondary antibody (antirabbit CF 488) and subsequently examined using CLSM.

### 2.9 Statistical Analysis

Statistical analysis was performed using the Mann–Whitney *U* test to identify differences between treatment groups.^42,43^ Confidence intervals were calculated, and the data are presented as the mean ± standard deviation. Significance was determined through statistical analysis, with a significance level set at a *p* value below 0.05.

## 3 Results

### 3.1 The Effect of DEHP on Cell Viability, Wound Healing, and the Expression of Malignant-Related Proteins

**Figure 2:**
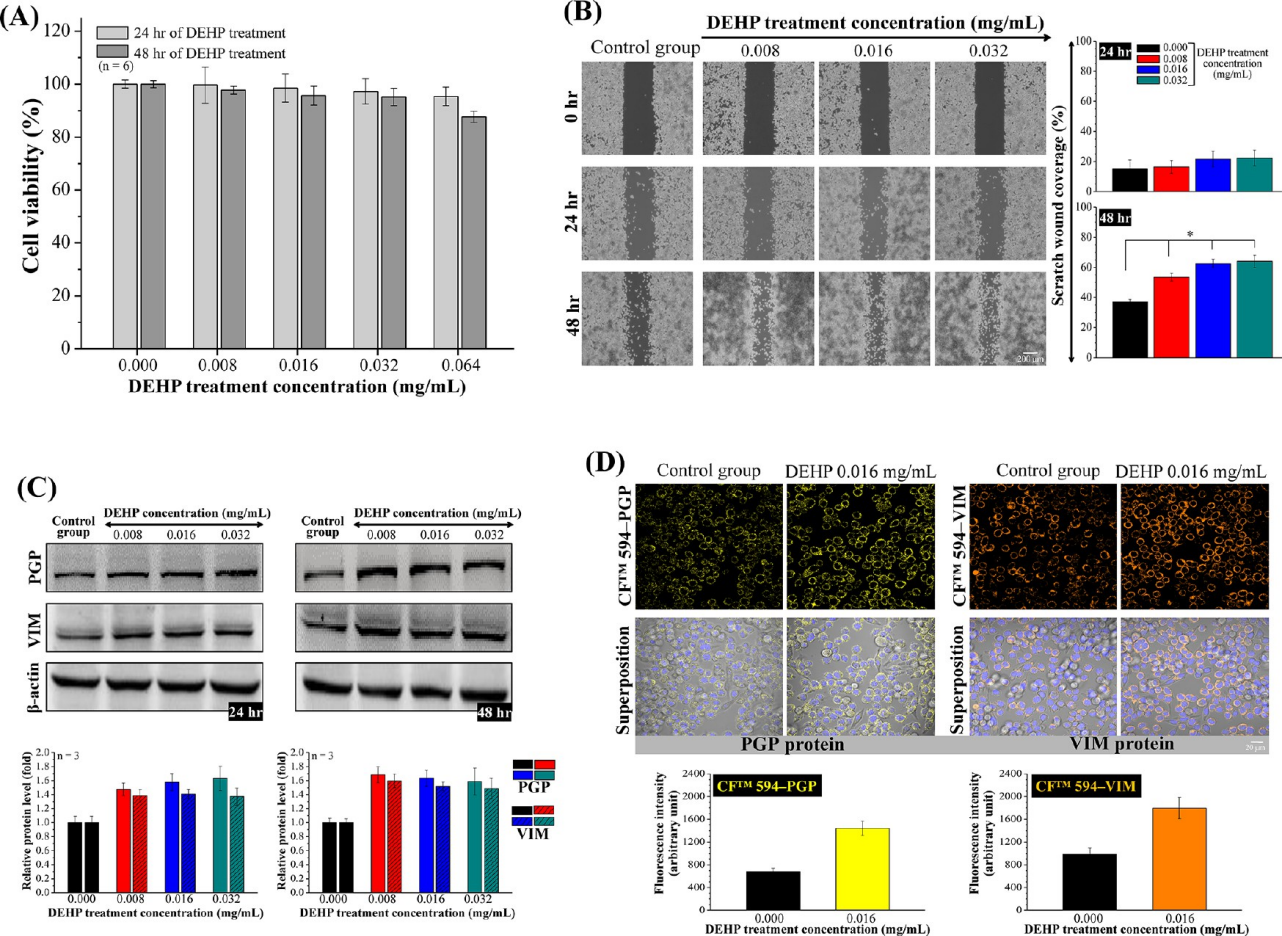
DEHP exhibited a notable capacity to promote gastric cancer cell migration and increase the expression of malignant-associated proteins. (A) Assessment of MNK45 cell viability in response to varying DEHP concentrations. (B) Evaluation of the migration ability of MKN45 under DEHP treatment for 24 or 48 h. (C,D) Quantification of PGP and VIM protein expression levels along with corresponding fluorescent signals in DEHP-treated MKN45 cells. *Significant differences at *p* < 0.05 compared with the untreated group.

We assessed the effect of various concentrations (0.000, 0.008, 0.016, 0.032, and 0.064 mg/mL) of DEHP on gastric cancer cell viability using the MTT assay. MKN45 cell viability remained unaffected when exposed to DEHP solutions below 0.032 mg/mL, but a slight decline in viability was observed after 48 h of treatment with 0.064 mg/mL DEHP (Figure 2A). As a result, we treated the cells with DEHP solutions ranging in concentration from 0.000 to 0.032 mg/mL in subsequent experiments. DEHP has been reported to promote cell proliferation, migration, and inflammatory response. We evaluated the effect of DEHP on wound healing abilities in MKN45 cells. Scratch distances and wound closure were determined to assess cell migration by comparing images taken at times of 0–48 h. Our results demonstrated an increase in cell migration and wound coverage after the DEHP treatment. Specifically, the scratch wound coverage in the wound healing assay was 37.04 ± 1.77, 53.56 ± 2.56, 62.59 ± 2.85, and 63.95 ± 4.16% for DEHP treatment concentrations of 0.000, 0.008, 0.016, and 0.032 mg/mL, respectively (Figure 2B). We studied the potential regulatory effect of DEHP on malignancy in gastric cancer cells and analyzed the associated protein expression by Western blotting. Treatment with DEHP concentrations ranging from 0.008 to 0.032 mg/mL for 24 h significantly increased the levels of PGP from 1.47 ± 0.09 to 1.63 ± 0.17 and those of VIM from 1.37 ± 0.12 to 1.41 ± 0.06 compared to the control group (set as 1.00; three independent experiments each) (Figure 2C). The group treated with 0.016 mg/mL DEHP exhibited significantly higher protein expression, as evidenced by a 2.12-fold increase in PGP fluorescence intensity and a 1.81-fold increase in VIM fluorescence intensity, surpassing the levels observed in the untreated group (Figure 2D).

### 3.2 Preparation and Characterization of the ACS and TFD Polymers

**Figure 3:**
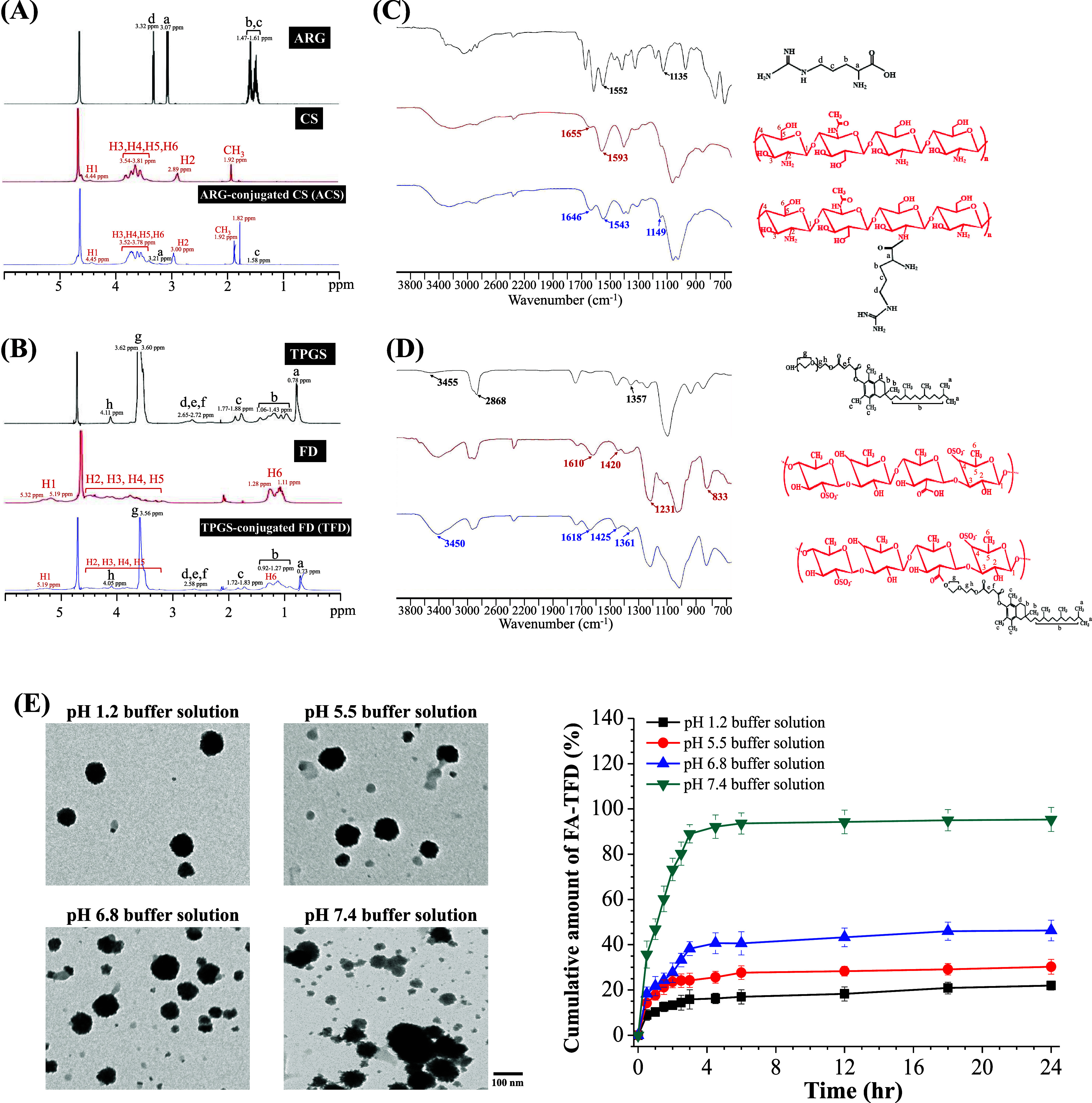
Characteristics and release profile of ACS/TFD NPs. (A–D) NMR and FTIR analyses were conducted on four distinct materials (ARG, CS, TPGS, and FD) and both components of the NPs (ACS and TFD). (E) The morphologies and release profiles of ACS/TFD NPs were examined across various pH conditions, including the simulated gastric fluid (pH 1.2), simulated gastric mucosa (pH 5.5), and simulated extracellular and intracellular tumor environments (pH 6.8 and pH 7.4).

The ACS polymer is composed of a cationic CS main chain and an attached ARG side chain. Distinct signals were observed in the CS ^1^H NMR spectrum, including a peak at δ 1.92 ppm representing three methyl H atoms (acetyl-glucosamine GlcNAc), a signal at δ 2.89 ppm originating from H2 (glucosamine, GlcN), a series of overlapping signals from δ 3.54–3.81 ppm, indicating the presence of H3–H6, which are connected to the nonanomeric C3–C6 carbons in the glucopyranose ring, and a peak at δ 4.44 ppm arising from H1 in the anomeric proton. A proton chemical shift occurred at 1.58 ppm, which was attributed to the combined proton intensity provided by the β- and γ-carbons of ARG, falling within the 1–2 ppm range. The protons from the δ-carbon of ARG were integrated into the CS backbone at 3.21 ppm (Figure 3A). Furthermore, when the spectra of the ACS samples were compared with those of ARG and CS, significant changes were observed in the FTIR spectra. The guanidine group exhibited a distinct band at 1543 cm^–1^, and the C–C–N asymmetric bend displayed a prominent band at 1149 cm^–1^. Moreover, a new band appeared around 1646 cm^–1^, indicating the presence of an amide bond linking the CS and ARG (Figure 3C). Based on a comprehensive analysis of the ACS NMR and FTIR spectra, we inferred that ARG was effectively grafted onto the CS amino groups. TPGS exhibited a structure with amphiphilic properties comprising a lipophilic alkyl tail and a hydrophilic polar head and featured a water-soluble PEG1000 portion as well as a fat-soluble α-tocopherol portion. Distinct signals were observed in the TFD ^1^H NMR spectrum (Figure 3B). Distinct signals were observed in the TFD ^1^H NMR spectrum. In the ^1^H NMR spectrum of TPGS, signals corresponding to the ethylene protons of the PEG chain were observed at δ 3.60 and 3.62 ppm. The signal at δ 2.65–2.72 ppm was assigned to the −CH_2_CH_2_ moiety of the TPGS succinyl group, whereas those in the aliphatic region (δ 0.78–1.43 ppm) were attributed to various protons in the vitamin E-tail. In the NMR spectra of the TFD, the ethylene protons of the PEG chain shifted to a peak of 3.56 ppm, while anomeric H1 signals in the FD were detected at 5.19 ppm. These results indicate that the characteristic FTIR peaks at 1357 cm^–1^ correspond to the PEG −CH_2_ group, whereas those at 2868 and 3455 cm^–1^ correspond to a −CH stretch band and the terminal hydroxyl group −OH in the TPGS molecule, respectively. The peaks observed at 1610 and 1420 cm^–1^ (corresponding to the COOH stretching vibrations), a band around 1231 cm^–1^ (attributed to S=O stretching), and a small band at 833 cm^–1^ (indicating C–O–S bending) were assigned to the FD molecule. Finally, analysis of the chemical structure of the synthesized TFD polymer revealed distinct peak shifts, particularly at 3450 cm^–1^, corresponding to the hydroxyl group (−OH) in TPGS. Additionally, the C–O symmetric and C=O asymmetric stretching, attributed to the carboxyl groups on FD, exhibited shifts at 1425 and 1618 cm^–1^, respectively (Figure 3D). These findings suggest that the binding of TPGS to the FD was successful during conjugation.

### 3.3 Characterization of ACS/TFD NPs and Drug Release Profiles

The NPs were formed through ionic gelation by combining positively charged ACS with a negatively charged TFD. Table 1 shows that varying weight proportions of ACS:TFD (0.25:0.50, 0.50:0.50, 1.00:0.50, 1.50:0.50, and 2.00:0.50 mg/mL) resulted in nanoscale complexes with mean particle sizes ranging from 200 to 450 nm and positive zeta potential values. The excess positively charged ACS molecules led to entanglement on the NP surface. Among the formulations, an ACS/TFD ratio of 1.50:0.50 mg/mL exhibited the smallest particle size (193.06 ± 7.73 nm), a desirable positive zeta potential value (30.32 ± 0.34 mV), and efficient TFD complexation (48.96 ± 3.13%) of the ACS/TFD NPs. Consequently, NPs with this specific composition were selected for subsequent investigation. Evaluation of the pH stability and release profile of the ACS/FA*–*TFD NPs under simulated gastric fluid conditions (pH 1.2 with pepsin) revealed a stable spherical morphology within the matrix structure, with TFD constituting 21.96 ± 1.98% of the release within 24 h. The NPs’ morphology was similar to that observed in deionized water at pH 5.5 (representing gastric mucosa), and the percentage of TFD released from the NPs was 30.25 ± 3.26%. In contrast, exposure to a buffer solution with a pH range of 6.8 to 7.4, which simulated extracellular and intracellular tumor tissue conditions, caused partial deprotonation of the ACS of −NH_3_^+^ groups. As a result, the electrostatic interactions between ACS and TFD weakened, leading to slight destabilization of the structural conformation of the NPs. The percentage of TFD released within the initial 6 h ranged from 40.58 ± 5.15% at pH 6.8 to 93.58 ± 4.68% at pH 7.4 over a 24 h period, with values reaching 46.29 ± 4.56% at pH 6.8 and 95.32 ± 5.32% at pH 7.4 (Figure 3E).

**Table 1:** Effect of Different ACS/TFD Proportions on Particle Sizes, Polydispersity Indices, and Zeta Potential Values of the Prepared ACS/TFD NPs (*n* = 5)^a^

**ACS:TFD(**mg/mL)	**mean particle size (nm)**	**polydispersity indices**	**zeta potential value (mV)**
0.25:0.50	445.14 ± 74.82	0.48 ± 0.15	–23.31 ± 5.63
0.50:0.50	325.06 ± 20.96	0.37 ± 0.03	24.53 ± 3.42
1.00:0.50	235.25 ± 6.18	0.28 ± 0.08	29.43 ± 0.52
1.50:0.50	193.06 ± 7.73	0.24 ± 0.02	30.32 ± 0.34
2.00:0.50	205.36 ± 8.47	0.35 ± 0.04	33.63 ± 2.15

a ACS, arginine-conjugated chitosan; TFD, d-α-tocopherol polyethylene glycol succinate-conjugated fucoidan; NPs, nanoparticles.

### 3.4 The Effect of TFD in the TFD Solution or ACS/TFD NPs on Cell Motility and Viability

**Figure 4:**
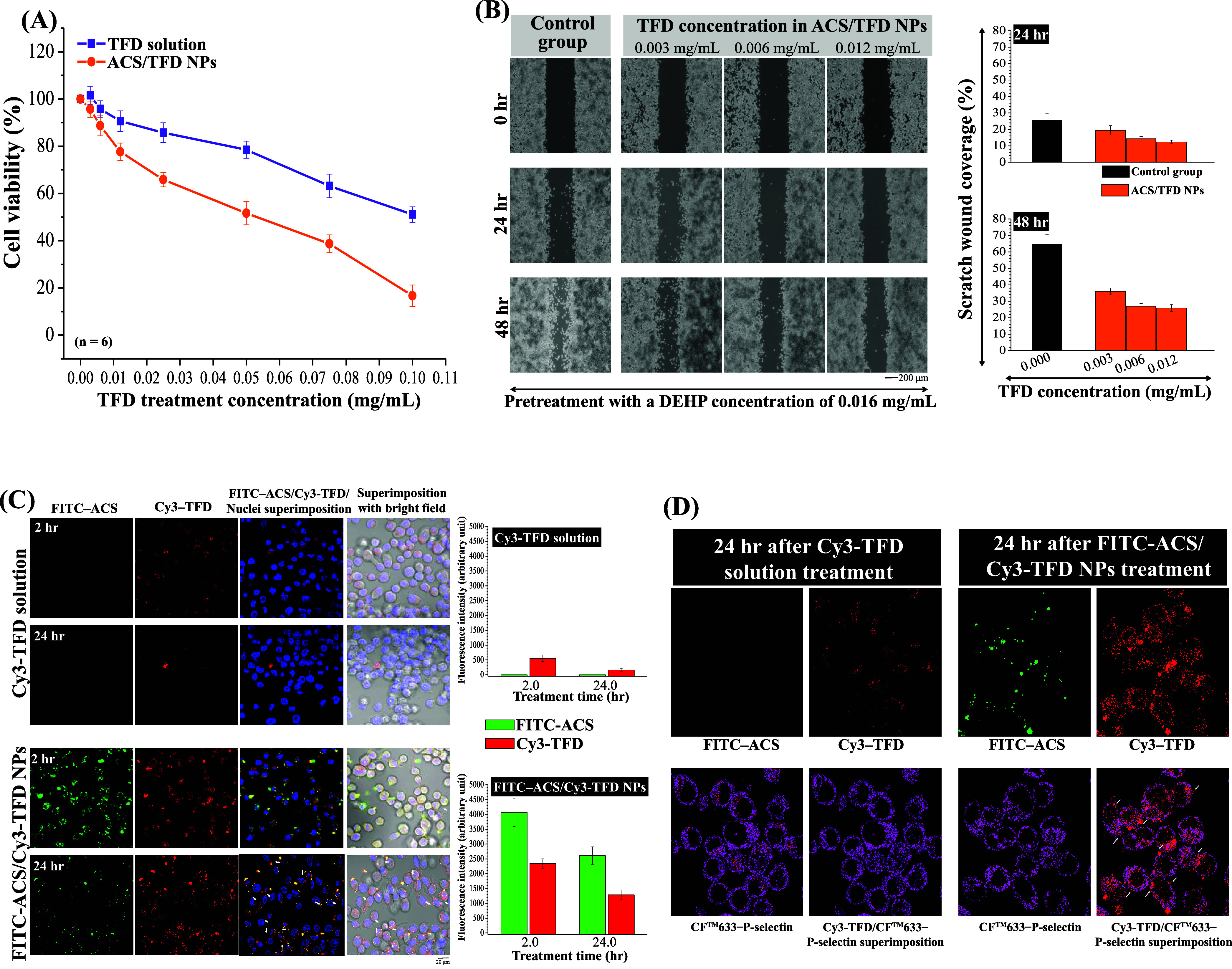
Assessment of the *in vitro* functionality and cellular distribution of ACS/TFD NPs. (A) Evaluation of MKN45 cell viability subsequent to treatment with TFD solution or ACS/TFD NPs. (B) Investigation into the impact of TFD within ACS/TFD NPs on the migratory behavior of DEHP-treated MKN45 cells. (C) Fluorescence images and quantitative analysis of MKN45 cellular uptake after incubation with either Cy3–TFD solution or FITC–ACS/Cy3–TFD NPs for 2 or 24 h. (D) Examination of the P-selectin targeting capabilities of Cy3–TFD solution or FITC–ACS/Cy3–TFD NPs in MKN45 cells, with white arrows denoting colocalization of CF633-P-selectin and Cy3–TFD.

TPGS is widely used in drug delivery systems and has demonstrated an apoptogenic activity against various types of cancers. Here, we evaluated the efficacy of TFD in the TFD solution and ACS/TFD NPs on MKN45 cell viability following DEHP treatment. Figure 4A shows that cell viability of the TFD solution group ranged from 85.75 ± 4.17 to 51.07 ± 3.25% at concentrations of 0.025–0.100 mg/mL. The cell survival rate after treatment with ACS/TFD NPs was noticeably lower than that of the TFD solution. The anticancer activity of ACS/TFD NPs (TFD concentrations of 0.006–0.050 mg/mL) on MKN45 cells was significantly higher than that of the TFD solution. Moreover, we treated normal cells (NIH/3T3) with various concentrations of ACS, TFD, and ACS/TFD NPs. Notably, the viability levels observed in the treated normal cells under different conditions were consistent, suggesting that ACS was noncytotoxic. We also evaluated the inhibitory effects of TFD or ACS/TFD NPs on the normal cell viability. We observed a slight decline of approximately 15% in viability at TFD concentrations of 0.10 mg/mL (Supporting Information, Figure S1). FD has an affinity for P-selectin and suppresses gastric cancer cell growth, migration, and invasion. Cell migration was examined by a wound healing assay to confirm the function of the TFD in the TFD solution and ACS/TFD NPs (Figure 4B). When the treatment duration was extended to 48 h, the presence of cells was partial when subjected to a low concentration of TFD (0.003 mg/mL), whereas the control group (without treatment) exhibited complete filling of the scratched area. Furthermore, migration was quantified at specific time points following scratching and revealed a decrease in cell migration in the presence of TFD. The degree to which migration was inhibited appeared to be directly correlated with TFD concentrations ranging from 0.006 to 0.012 mg/mL, suggesting suppressed cell migration without any effect on cell growth.

### 3.5 Cellular Distribution and an *In Vitro* Study of Cancer-Targeting ACS/TFD NPs

To investigate the distribution of Cy3-conjugated TFD molecules or FITC–ACS/Cy3–TFD NPs within MKN45 cells, we used confocal laser scanning microscopy (CLSM) and MetaMorph software to quantify FITC–ACS (green spots) and Cy3–TFD (red spots) fluorescence intensities. The fluorescence signals of the incubated NPs remained intact (indicated by superimposed green/red spots, i.e., white spots and white arrows in Figure 4C) after being internalized into the intercellular space and cell cytoplasm for 2 h. As the incubation time was prolonged to 24 h, the superimposed images revealed fewer green spots (FITC–ACS) and white spots (FITC–ACS/Cy3–TFD) in the perinuclear space, indicating that the NPs within the cellular spaces were no longer intact. The cells treated solely with Cy3–TFD exhibited less pronounced fluorescence signals in the intercellular spaces than did the group treated with fluorescent NPs. The quantitative analysis of the data demonstrated that the Cy3-FD fluorescence intensity in the NP group increased by 4.22-fold and 7.93-fold after 2 and 24 h of treatment, respectively, compared to the Cy3–TFD solution alone. Moreover, the CLSM analysis revealed the colocalization of CF633-P-selectin and Cy3–TFD, demonstrating an interaction with the cell surface P-selectin (indicated by red/purple spots and white arrows in Figure 4D) in MKN45 cells. This finding indicates that variations in the TFD delivery efficiency and absorption capacity were related to the TFD released by ACS/TFD NPs, successfully targeting the specific P-selectin protein. Moreover, our findings revealed that the binding capacity of TFD to P-selectin increases proportionally with the TFD dosage, which can be significantly decreased by adding a competitive P-selectin antibody (Supporting Information, Figure S2).

### 3.6 Interaction between DEHP and ACS/TFD NPs on the Hypothesized Signal Transduction Pathways and Metastasis-Related Proteins in MKN45 Cells

**Figure 5:**
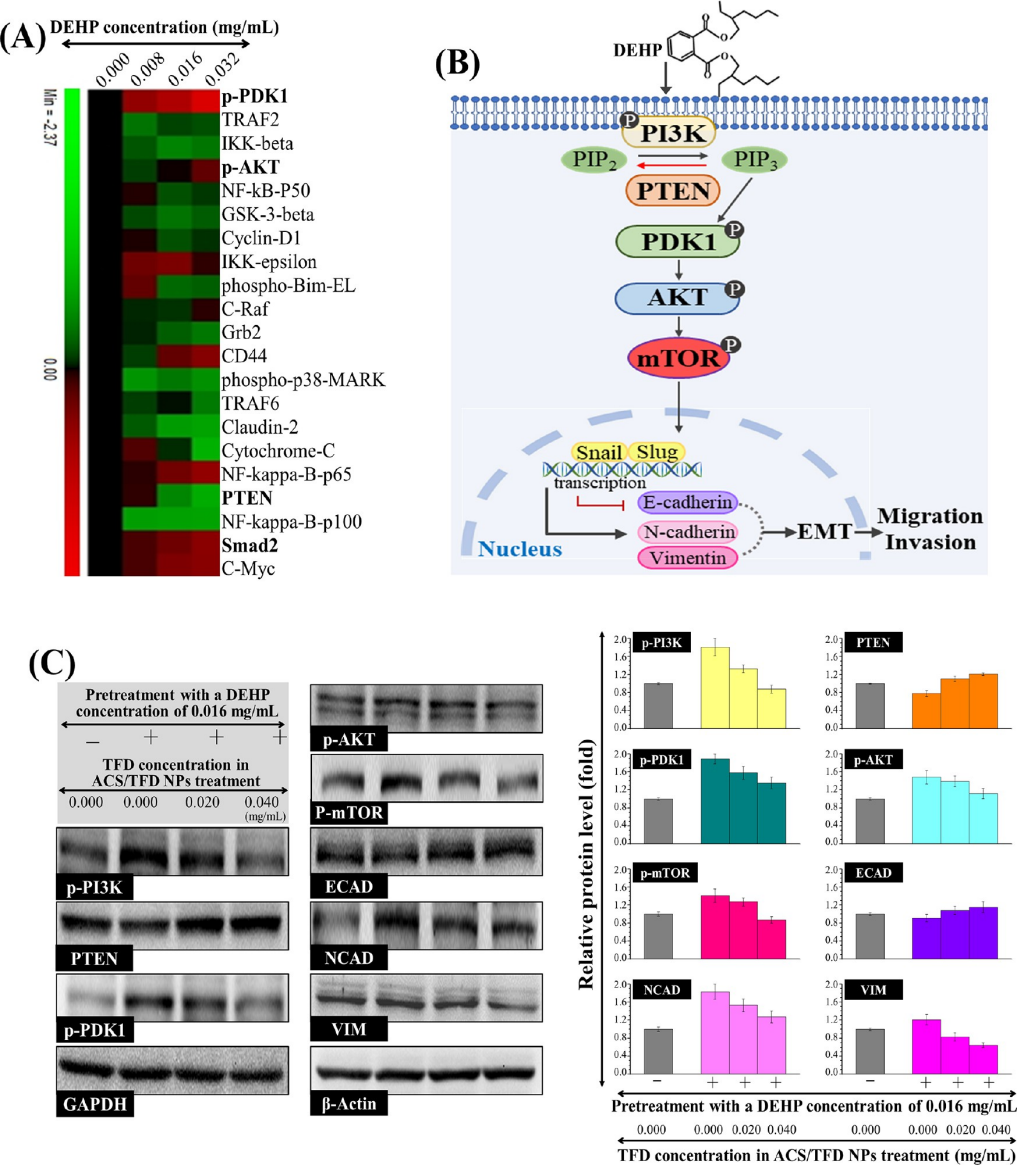
ACS/TFD NPs orchestrated alterations in the DEHP-triggered malignancy-associated signaling pathway. (A) The heat map represents the protein profiles of MKN45 cells influenced by diverse concentrations of DEHP treatment. (B) An envisioned signaling pathway potentially induced by DEHP was proposed. (C) ACS/TFD NPs exerted modulation over metastasis-related proteins in DEHP-treated MKN45 cells, encompassing p-PI3K, PTEN, p-PDK1, p-AKT, p-mTOR, ECAD, NCAD, and VIM.

As DEHP-treated MKN45 cells may exhibit distinct protein profiles related to metastasis, we conducted MWA to verify our hypothesis. The heat map analysis revealed the downregulation of PTEN expression, contrasting with the upregulation of phospho-PDK1 (p-PDK1), phospho-AKT (p-AKT), and Smad2 expression levels compared to the untreated control group (Figure 5A). We propose a potential signaling pathway by which DEHP induces the motility of gastric cancer cells (Figure 5B). DEHP treatment triggered a significant interaction between DEHP and PI3K in MKN45 cells, resulting in substantial downstream effects, including downregulation of PTEN and upregulation of PDK1, a crucial upstream regulator involved in activating AKT. Western blot analyses were performed on MKN45 cells to investigate whether the expression of associated proteins induced by DEHP was mediated through the PI3K/AKT/mTOR pathway. The data in Figure 5C indicate that pretreatment with DEHP (0.016 mg/mL) increased the phosphorylation levels of PI3K, PDK1, AKT, and mTOR, accompanied by a decrease in the level of PTEN expression within the system. Subsequently, band densities were quantified to assess the effect of the ACS/TFD NPs on promoting EMT by modulating the PI3K/AKT/mTOR pathway in DEHP-treated MKN45 cells. Treatment with ACS/TFD NPs (TFD concentrations of 0.020 to 0.040 mg/mL) consistently changed the EMT by decreasing NCAD and VIM expression while increasing ECAD expression. Additionally, the ACS/TFD NPs downregulated the phosphorylation of PI3K, PDK1, AKT, and mTOR in DEHP-pretreated MKN45 cells, suggesting their potential to inhibit the PI3K/AKT/mTOR pathway and suppress the EMT, including Smad2 signaling and the expression of mesenchymal markers, such as NCAD and VIM (Figure 5C).

### 3.7 Assessment of Antitumor Activity through Tumor Bioluminescence and Analysis of NP Distribution within the Tumor

**Figure 6:**
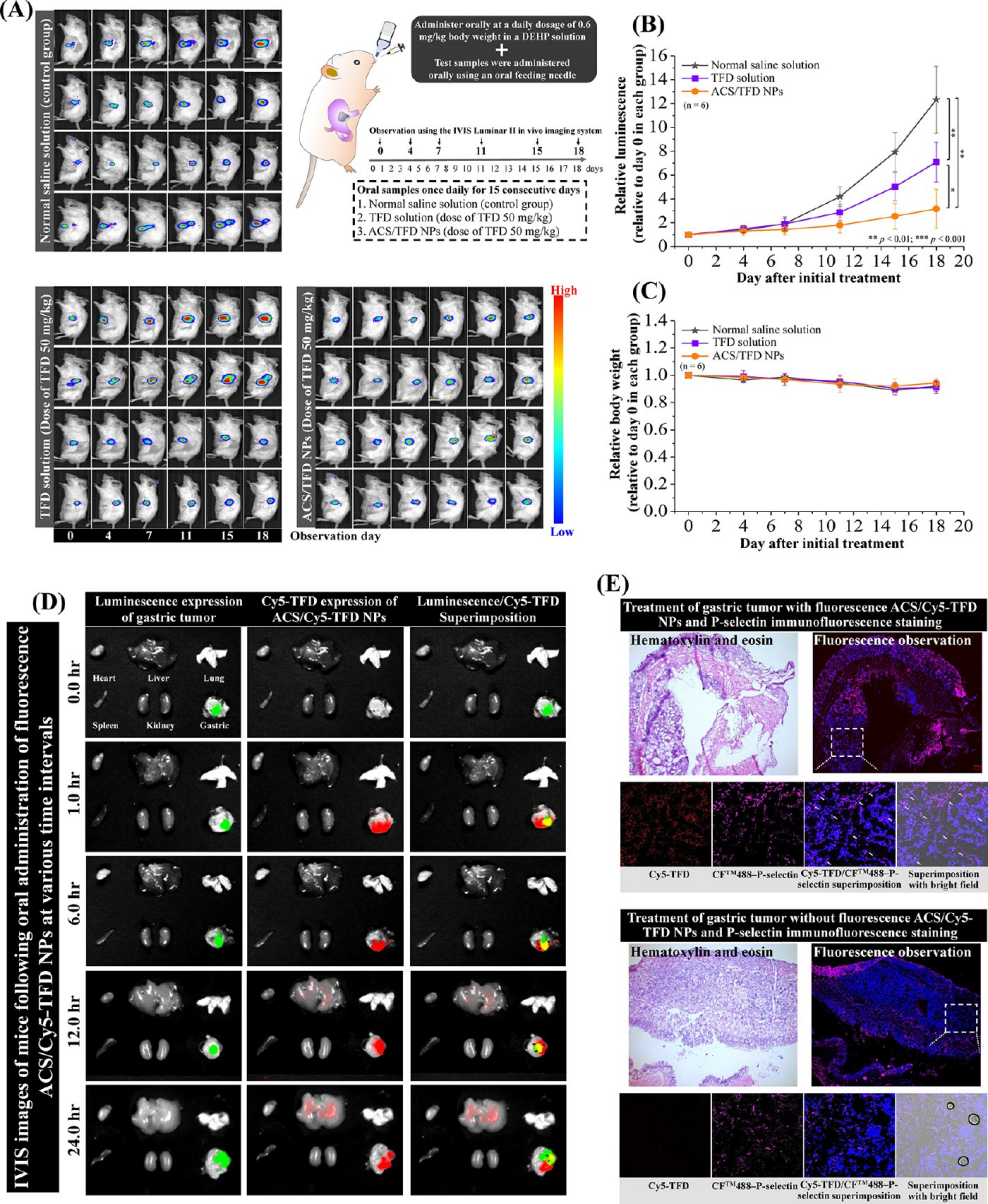
Assessment of the antitumor effects of diverse test samples was conducted within an orthotopic gastric tumor model. The mice were categorized into three groups, each consisting of six mice, and subjected to treatment with normal saline solution (control group) (stars), TFD solution (squares), or ACS/TFD NPs (circles). (A) Bioluminescence images of tumors in each treatment group were captured using an IVIS Luminar II *in vivo* imaging system at designated time points. (B) The relative luminescence signals emanating from tumors within each group were calculated at the specified time points. (C) Alterations in relative body weight were monitored over time. (D) The distribution of ACS/Cy5–TFD NPs in organs was analyzed post-treatment at different intervals (0, 1, 6, 12, and 24 h). The left side shows images of bioluminescence expression illustrating the gastric tumor, the middle portion displays fluorescence expression depicting NP distribution, and the right side presents the superimposition signals of bioluminescence and fluorescence. (E) Immunofluorescence staining of P-selectin within the gastric tumor tissue was performed on mice treated with fluorescence ACS/Cy5–TFD NPs via esophageal administration using an oral feeding needle at 24 h post-treatment and analyzed by CLSM imaging. *Significant differences at *p* < 0.05 and ** *p* < 0.01.

We established orthotopic luciferase-expressing gastric carcinoma in SCID mice to investigate antitumor activity *in vivo* and TFD-specific delivery of ACS/TFD NPs compared to the TFD alone solution. In the normal saline solution group, the bioluminescence signals of the gastric tumors increased significantly by 12.32 ± 2.79-fold over time (Figure 6A,B). In the ACS solution group, there was a trend of the tumor growth rate, accompanied by an increase in bioluminescence, reaching 8.65 ± 1.93-fold by day 18 (Supporting Information, Figure S3). In contrast, the tumor growth rate decreased, accompanied by an increase in bioluminescence (7.09 ± 1.66-fold) in the TFD solution group. Importantly, the ACS/TFD NP treatment inhibited tumor growth the most and exhibited a lower relative photon flux (3.16 ± 1.67-fold) compared to those of the other treatment groups of mice (Figure 6A,B). No significant changes in the average weight percentage were observed throughout the treatment period (Figure 6C). To further clarify the localization of TFD in different organs, fluorescent ACS/Cy5-conjugated TFD NPs were administered orally through the esophagus. The Cy5–TFD fluorescence signal was monitored at various time points using an *in vivo* optical imaging system technique. Figure 6D demonstrates that the stomach exhibited persistent fluorescent signals, and colocalization was observed between the fluorescent and bioluminescent gastric tumor signals (indicated by superimposed green/red spots and black arrows). The tumor’s Cy5–TFD fluorescence intensities ranged from 3,430,000 to 5,520,000 over 1–24 h. Compared with the 1 h group, the relative fluorescence intensities were 1.19-fold, 0.86-fold, and 0.74-fold at 6, 12, and 24 h, respectively. Following a 24 h administration of fluorescent ACS/Cy5–TFD NPs, tissue sections were subjected to immunofluorescence staining using an anti-P-selectin antibody to identify the interaction between TFD and its associated proteins expressed on gastric cells via CLSM observations. Cy5–TFD fluorescence signals (red dots) specified the colocalization and interaction between TFD and P-selectin in the gastric tumor tissue (indicated by superimposed red/purple spots and white arrows). This observation, depicted in Figure 6E, confirms the release of TFD by ACS/TFD NPs and their ability to target tumors in an orthotopic gastric tumor mouse model.

### 3.8 Immunohistochemistry Analysis of the Gastric Tumor and Adjacent Organs

**Figure 7:**
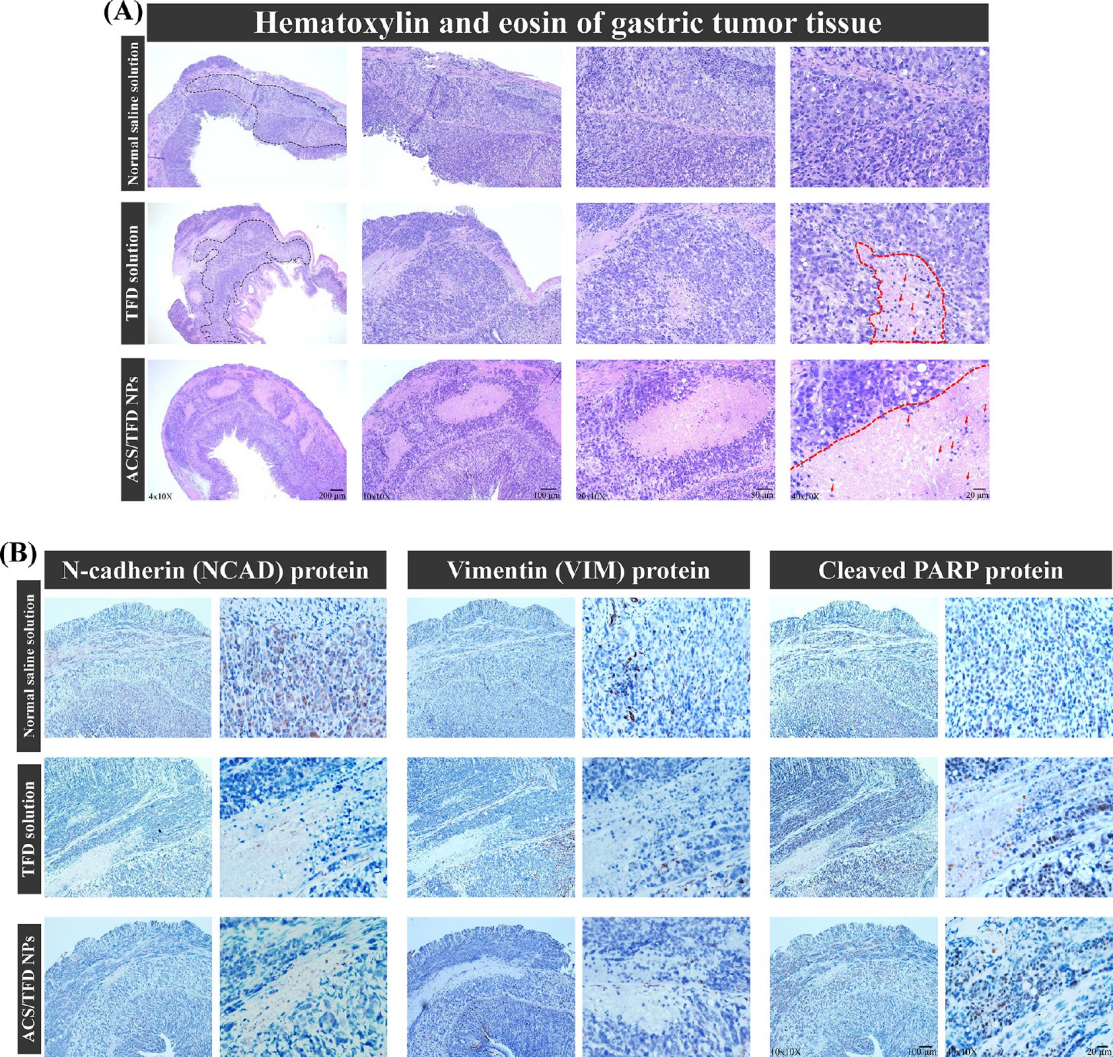
Histological and immunohistochemical evaluations of gastric tumors. (A) Tumors from the three treatment groups were examined with hematoxylin and eosin staining, with the black frame indicating the gastric antrum tissue. Necrotic areas and granular eosinophilic cells were marked by red lines and red arrows, respectively. (B) Immunohistochemical staining was performed for NCAD, VIM, and cleaved PARP on tumors from three treatment groups, with augment protein expression denoted by coffee-colored dots.

Gastric tumor tissue biopsies from the experimental mice were stained with hematoxylin and eosin for histological analysis. In Figure 7A, the gastric antrum tissue of the mice treated with either a normal saline solution or a TFD solution displayed significant malignant clusters of epithelial cells within the gastric wall at a 40× magnification (black frame). A substantial population of granular eosinophilic cells had extended within the muscularis propria under the normal saline solution treatment at a 400× magnification and was expressed within the mucosal layer (indicated by red arrows). Furthermore, mice treated with ACS/TFD NPs exhibited grade 2 tissue necrosis, characterized by more than two-thirds loss of cancer cells and increased tumor necrosis (right of the red line). Immunohistochemistry was performed to validate the *in vitro* findings and to evaluate the effects of the ACS/TFD NP treatment on metastasis and apoptosis by assessing mesenchymal markers (NCAD or VIM) and cell apoptosis (cleaved PARP). Figure 7B shows significant decreases in NCAD and VIM expression and an increase in cleaved PARP expression (coffee dots) in the tumors after treatment.

**Figure 8:**
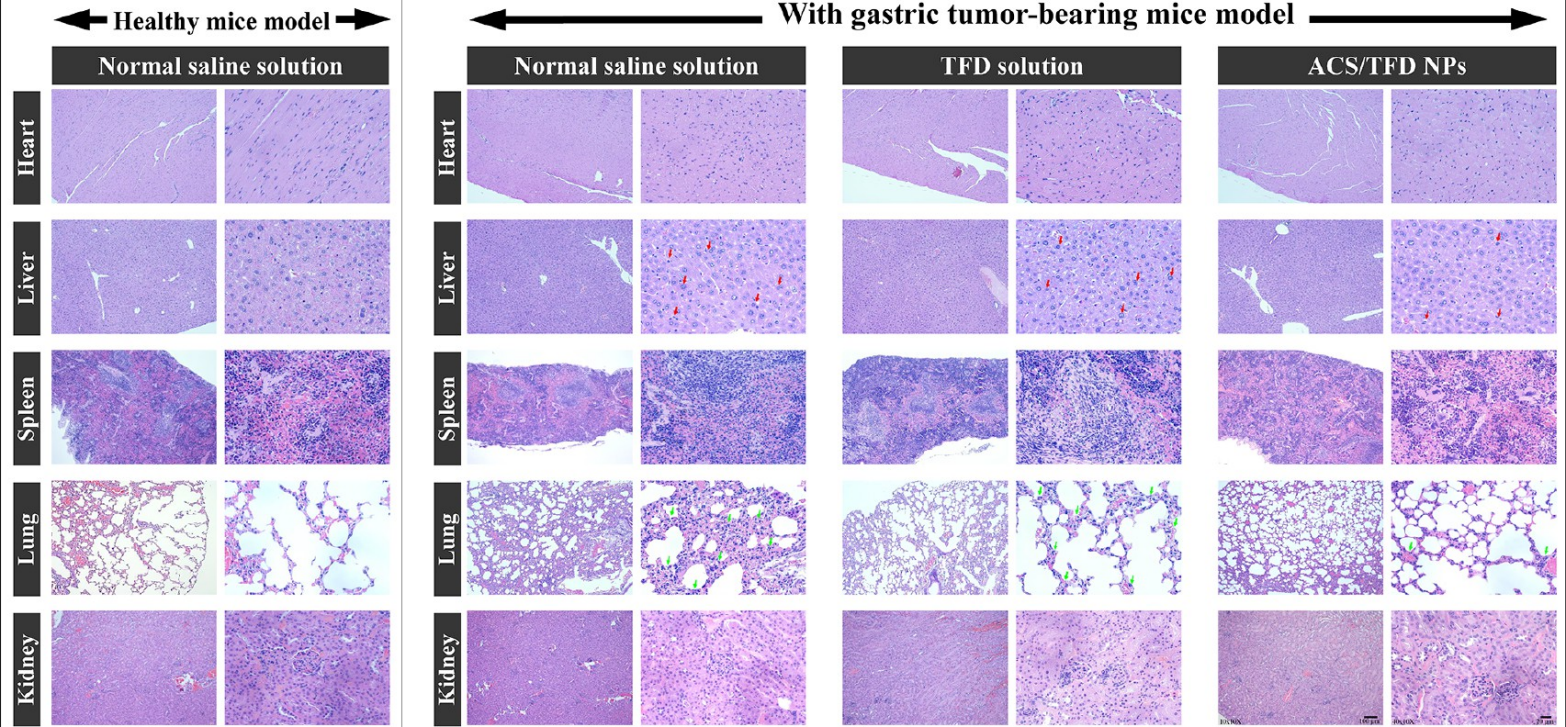
Concluding the treatment regimen, diverse organ biopsies in normal and gastric tumor mice from distinct treatment groups, including normal saline solution, TFD solution, and ACS/TFD NPs, underwent histological analysis, employing hematoxylin and eosin staining. Thickened interstitial pulmonary edema and neutrophil infiltration were indicated by green arrows and red arrows, respectively.

Furthermore, heart, liver, spleen, lung, and kidney specimens were subjected to hematoxylin and eosin staining for the observations (Figure 8). The lung tissue from the normal saline group exhibited inflammatory cell exudation, scattered red blood cells present in multiple alveolar spaces, and thickened interstitial pulmonary edema (green arrows). The ACS/TFD NP-treated group exhibited markedly reduced pathological damage, displaying a clearer alveolar structure and diminished signs of inflammation, closely resembling the lung structure observed in the healthy mouse model. The density of neutrophil infiltration and swelling hepatocytes was lower in the liver tissue biopsies in mice treated with ACS/TFD NPs than in those treated with normal saline solution (red arrows). These results indicate a correlation between the antitumor effects of ACS/TFD NPs, reduced metastasis, increased apoptosis, and a subsequent decrease in the inflammatory response during DEHP treatment for gastric carcinogenesis. Importantly, since safety is crucial for drug development, our findings revealed no significant changes in the average body weight percentage throughout the treatment. Furthermore, histological examination for safety verification demonstrated that ACS/TFD NPs did not induce any damage to major organs, including the stomach, heart, liver, spleen, lung, and kidney, compared with healthy mice (Supporting Information, Figure S4).

## 4 Discussion

Gastric cancer was the world’s fifth most common malignancy in 2020, with about 1.1 million new cases, and the fourth leading cause of cancer-related mortality, with about 800,000 deaths.^44−46^ Interestingly, chronic inflammation, such as gastritis and gastric ulcers, is thought to play a role in the development of gastric cancer. The US Environmental Protection Agency has set an oral reference dose (RfD) of 20 μg/kg body weight per day, while the European Union has established a tolerable daily intake (TDI) of 50 μg/kg of body weight per day for DEHP; both are predictable doses that do not cause significant adverse effects in human populations over the lifetime.^5,47^ Through the utilization of the body surface area normalization method to extrapolate animal doses to human doses, our study provides the foundation for establishing reference values, such as the RfD and TDI.^48^ Our findings revealed that oral administration of a DEHP solution to an orthotopic gastric tumor mouse model resulted in a notable increase in PGP protein expression levels (1.86 ± 0.31) and VIM (1.47 ± 0.16) compared to the control group set to 1.0 (Figure 1). To ensure compliance with safety regulations, the EN ISO 3826-1 standard governs containers used for collecting blood components, setting a maximum limit of 0.15 mg/mL for extractable DEHP in a flexible PVC material.^49,50^ In our study, treatment with DEHP at remarkably low concentrations (0.008 to 0.032 mg/mL) significantly enhanced MKN45 cell migration (Figure 2B). Furthermore, our observations revealed a simultaneous significant upregulation of PGP and VIM protein expression in the DEHP-exposed groups, suggesting a correlation between these observed changes (Figure 2C,D).

Previous studies have demonstrated that DEHP binds to estrogen receptor α (ERα), leading to carcinogenic potential and the ability to influence cell proliferation and tumor metastasis.^51,52^ Studies are needed to fully understand the effects of DEHP on gastric cancer MKN45 cells expressing ERα and its specific role in influencing the EMT-related process. To clarify the mechanism underlying the observed effects of DEHP on cell migration, we investigated the levels of regulatory proteins and metastasis-related proteins using the MWA method (Figure 5). PTEN, which is a dual-function protein and lipid phosphatase, regulates the pathway by dephosphorylating phosphatidylinositol-3,4,5-trisphosphate (PIP3), which recruits AKT to the cell membrane for subsequent phosphorylation and activation by PIP3-dependent kinases.^53^ Activated AKT modulates downstream substrates to control essential biological processes, including cell survival, metabolism, proliferation, and growth.^53−55^ Our findings indicate that DEHP was correlated with downregulated PTEN protein levels and upregulated p-PDK1 and p-AKT levels, demonstrating a concentration-dependent effect of DEHP on these signaling molecules. Both the PI3K/AKT/mTOR and Smad2 signaling pathways are activated during the EMT, as indicated by the upregulation of mesenchymal markers, including NCAD and VIM.^56^ Western blot analysis was conducted on DEHP-treated MKN45 cells, confirming upregulation of PI3K, PDK1, AKT, and mTOR phosphorylation levels, accompanied by increased expression of NCAD and VIM, compared to that of the untreated control group. These results indicate an EMT-associated change induced by the DEHP treatment (Figure 5B,C).

Nanocarriers represent a promising platform for offering a valuable approach to modulating EMT-related pathways.^57^ Zhou et al. reported that codelivering doxorubicin and a transforming growth factor beta (TGF-β) receptor inhibitor via a nanocarrier effectively inhibits the TGF-β/Smad signaling pathway, resulting in suppressed EMT progression in breast cancer cells.^58^ Notably, the distinct pharmacokinetic profiles of different drug components can introduce complexities that may affect the synergistic effects of combined therapy *in vivo*.^59^ In our study, NPs were developed by combining ACS (ARG-conjugated CS) and TFD (TPGS-conjugated FD), and incorporating the amino acid ARG into ACS endowed the NPs with a net positive charge at pH 7.0.^60^ After acid hydrolysis of the ester bond in TPGS and TFD, the resulting solutions were analyzed by using LC–MS. TPGS, containing an ester bond, undergoes further hydrolysis to produce vitamin E.^61^ Quantification revealed that TPGS-FD contains a significant 25.7% (w/w) TPGS content (Supporting Information, Figure S5). The TPGS treatment stimulated the generation of reactive oxygen species, leading to the accumulation of cleaved PARP in the molecular pathway that facilitates apoptosis in hepatocellular carcinoma cells.^62^ FD interferes with the interaction between TGF-β1 and its receptor, resulting in a substantial decrease in the cellular response to TGF-β1, as evidenced by reduced phosphorylation of Smad2, a critical downstream signaling molecule in this pathway.^63^ Our developed ACS/TFD NPs induce partial TFD release within the extracellular tumor tissue, facilitated by the binding of TFD to P-selectin, a cell membrane protein. Simultaneously, the NPs effectively enabled significant TFD release within the intracellular tumor tissue, significantly attenuating DEHP-induced cell migration in gastric cancer MKN45 cells (Figure 4B,D). *In vivo* studies showed the superior efficacy of our NP delivery system, surpassing the free TFD solution, in targeting and treating gastric tumors, as evidenced by reduced tumor bioluminescence expression and downregulation of metastasis and apoptosis markers, including NCAD, VIM, and cleaved PARP (Figures 6 and 7). Furthermore, administering the NPs resulted in reduced levels of inflammation at nontumor tissue sites, further emphasizing the favorable attributes of this NP-based approach.

## 5 Conclusions

The present study revealed that an exceptionally low concentration of DEHP enhanced gastric cancer cell migration and promoted EMT by modulating the PI3K/AKT/mTOR and Smad2 signaling pathways in DEHP-treated MKN45 cells. Our initial TFD design, delivered via NPs, effectively bound to the P-selectin protein, leading to improved antigastric tumor capability and reduced expression of associated malignant proteins and highlighting the promising clinical therapeutic potential of our approach.

## Supporting Information Available

Additional experimental details; methods; extra figures related to the MTT assay, binding assay, assessment of the antitumor effects, and LC–MS assay; supplementary references (PDF)
