# Supplementary material for: Nanoparticles for Augmenting Therapeutic Potential and Alleviating the Effect of Di(2-ethylhexyl) Phthalate on Gastric Cancer
Source: ACS Appl Mater Interfaces. 2024 Apr 4;16(15):18285–99. doi: 10.1021/acsami.3c15976 (PMC11040586; doi:10.1021/acsami.3c15976)
Supplement: Supplementary file 1 — am3c15976_si_001.pdf [file am3c15976_si_001.pdf]

Supporting Information

Nanoparticles for Augmenting Therapeutic Potential and Alleviating the Effect of Di(2-Ethylhexyl) Phthalate on Gastric Cancer

Hau-Lun Huang[a#], Kuo-Wei Chen[b#], Hsiao-Wei Liao[a], Ling-Yu Wang[c], Shin-Lei Peng[d], Chih-Ho Lai[e], Yu-Hsin Lin[a,f,g]*

[a] Department of Pharmacy, National Yang Ming Chiao Tung University, Taipei 112304, Taiwan

[b] Division of Hematology and Oncology, Cheng Hsin General Hospital, Taipei 112401, Taiwan

[c] Institute of Pharmacology, National Yang Ming Chiao Tung University, Taipei 112304, Taiwan

[d] Department of Biomedical Imaging and Radiological Science, China Medical University, Taichung 40402, Taiwan

[e] Department of Microbiology and Immunology, Molecular Infectious Disease Research Center, Chang Gung University and Chang Gung Memorial Hospital, Taoyuan 33302, Taiwan

[f] Medical Device Innovation and Translation Center, National Yang Ming Chiao Tung University, Taipei 112304, Taiwan

[g] Department of Medical Research, China Medical University Hospital, China Medical University, Taichung 40402, Taiwan

***Correspondence to:**

Yu-Hsin Lin, Professor

Corresponding Author

Department of Pharmacy, National Yang Ming Chiao Tung University, 40402

Fax: 886-2-2823-2940

E-mail: ylhsin@nycu.edu.tw

[#] The first two authors (Hau-Lun Huang and Kuo-Wei Chen) contributed equally to this work

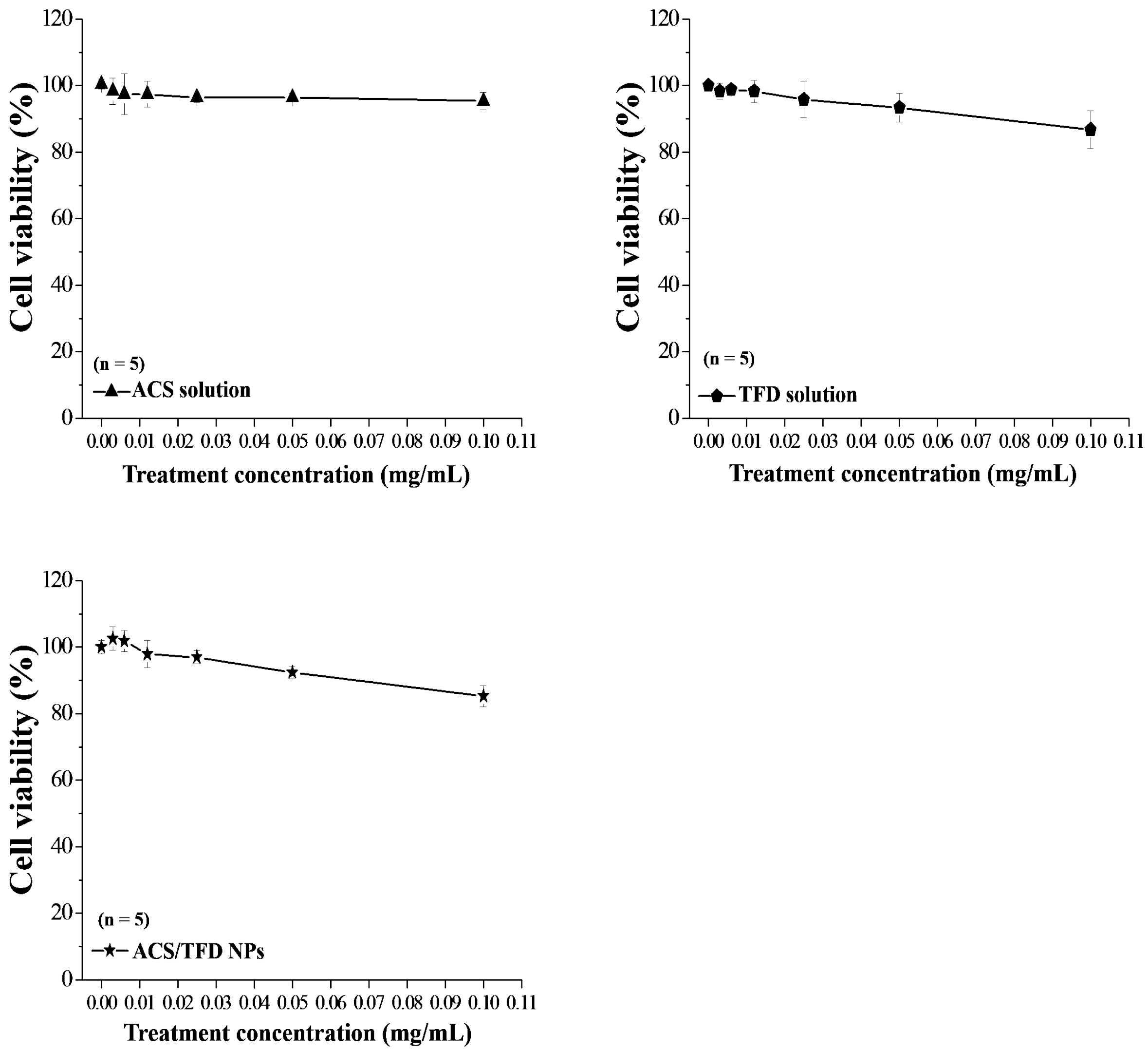

Figure S1. Cell viability of normal cells (NIH/3T3) was assessed using the MTT assay after treatment with various concentrations of ACS, TFD, and ACS/TFD NPs

Methods

Effects of TFD solutions on binding of P-Selectin

To assess the binding specificity of TFD with P-selectin, we employed a method involving human recombinant P-selectin (0.5 μg/50 μL) added to high-hydrophobicity 96 well plates and incubated overnight at 4°C. Following a PBS wash, the wells were blocked with 0.1 mL 3% bovine serum albumin for 1.0 hr. After another PBS wash, varying concentrations of Cy3-TFD solution were added to the wells for 1 hr, followed by three PBS washes. Fluorescence intensity was measured using a microplate spectrofluorometer with excitation and emission wavelengths set at 550 nm and 565 nm, respectively [1]. Simultaneously, an anti-P-selectin antibody (2.0 μg/mL) and a fluorescent test sample were introduced into the P-selectin-coated wells to assess the comparative binding specificity. The total volume of the resulting mixture in each well was 50 μL. After incubating for 1.0 hr, fluorescence measurements were conducted.

Results

The investigation revealed distinct fluorescence intensities for Cy3-TFD at concentrations of 100, 200, and 400 μg/mL, ranging from 660.95 ± 77.72 to 1759.35 ± 146.89. Notably, treatment with anti-P-selectin antibodies reduced the adhesion of TFD to targeting proteins by 40% due to the blockage of these antibodies. Our findings revealed that the binding capacity of TFD to P-selectin increases proportionally with the TFD dosage, which can be significantly decreased by adding a competitive P-selectin antibody (**Figure S2**).

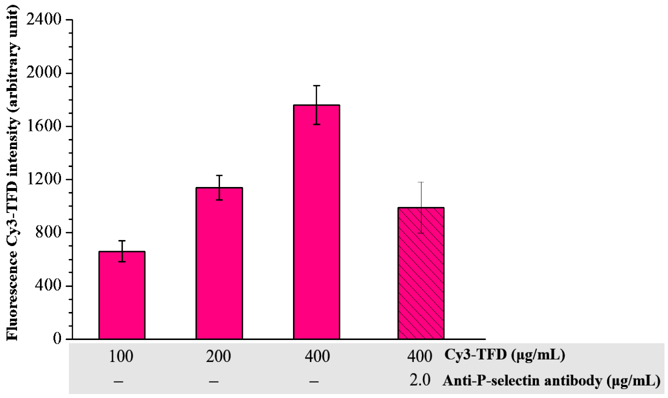

Figure S2. Binding assays of fluorescence Cy3-TFD to immobilized recombinant P-selectin

Reference

[1] Mamot, A.; Sikorski, P. J.; Siekierska, A.; de Witte, P.; Kowalska, J.; Jemielity, J. Ethylenediamine Derivatives Efficiently React with Oxidized RNA 3' Ends Providing Access to Mono and Dually Labelled RNA Probes for Enzymatic Assays and *in Vivo* Translation. *Nucleic Acids Res.* **2022**, *50*, e3, DOI: 10.1093/nar/gkab867.

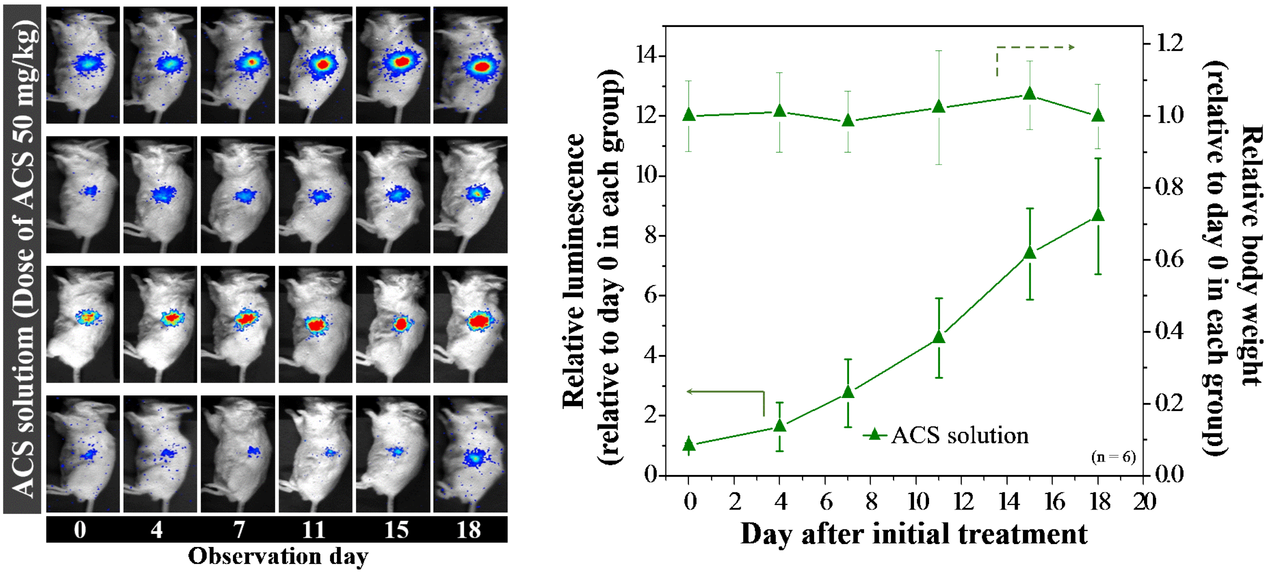

Figure S3. Assessment of the antitumor effects of the ACS solution on the orthotopic gastric tumor model through *in vivo* imaging system

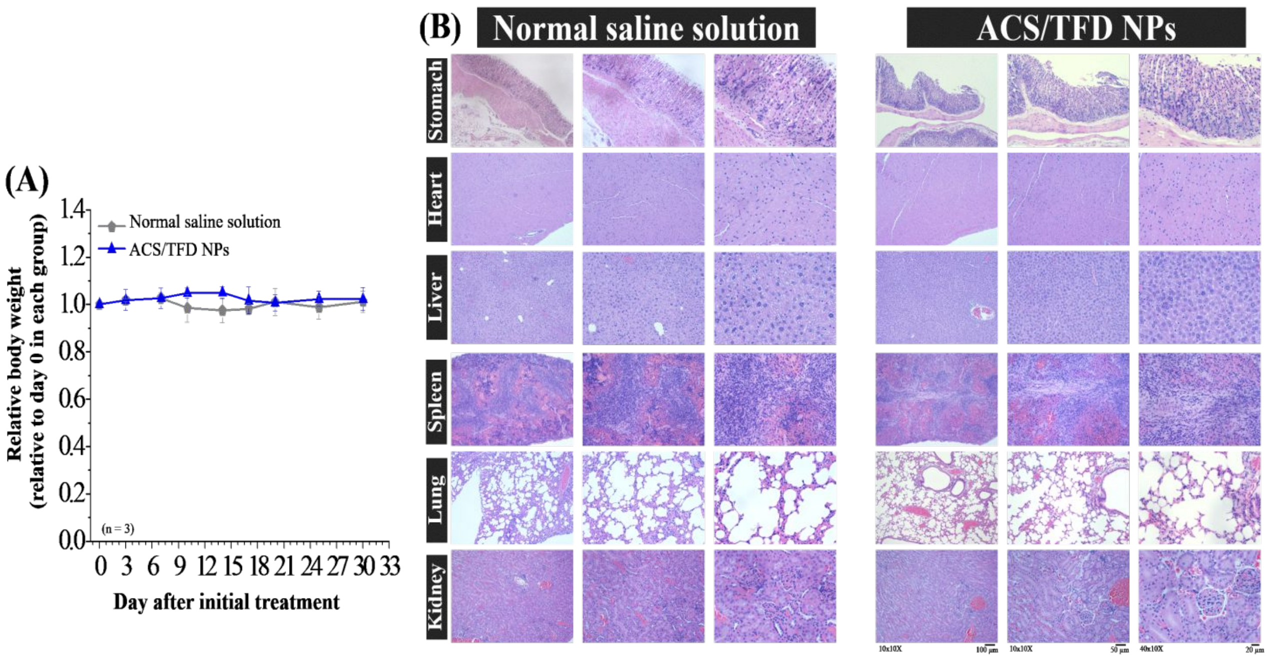

Figure S4. Changes in mice body weight and histological analysis of different organs in healthy mice after treatment with normal saline solution or ACS/TFD NP

Methods

Quantification of TPGS content in TFD using liquid chromatography-mass spectrometry

The TPGS-conjugated fucoidan (TPGS-FD or TFD) polymer was synthesized by integrating polymers with active terminal groups, involving the grafting of TPGS onto FD's carboxyl group via esterification [1,2]. Ester bonds exhibit slow hydrolysis under typical physiological conditions [3]. We utilize liquid chromatography–mass spectrometry (LC–MS) for a more accurate quantitative assessment of TFD. It is crucial to acknowledge that the cleavage of ester bonds may be expedited under strongly acidic conditions. To assess the impact of TPGS on TFD, we employed acid hydrolysis to liberate TPGS from TFD, following previous methods with slight modifications [3,4]. We combined 50 µL of 0.5 mg/mL TFD solution with 50 µL of 6M HCl. A standard 0.1 mg/mL TPGS solution was also included for testing. The samples were subjected to microwave irradiation (700 W) for 1 min using a household microwave oven (SAMPO, Taoyuan, Taiwan). Subsequently, the sample was cooled and dried in a SpeedVac vacuum centrifuge (miVac Duo Concentrator, GeneVac, UK). The residue was reconstituted in methanol for TPGS and TPGS-FD for subsequent analysis. As TPGS contains an ester bond and undergoes further hydrolysis during acid hydrolysis procedure, yielding vitamin E, we measured vitamin E as an alternative for TPGS quantification [5].

For the LC-MS analysis aimed at assessing vitamin E release, we employed an Agilent 1290 ultra-high-performance liquid chromatography (UHPLC) system (Agilent Technologies, Waldbronn, Germany) coupled with a Bruker maXis ultra-high-resolution (UHR)-time-of-flight (TOF) mass spectrometer (Bruker Daltonics, Bremen, Germany). Vitamin E released from a 100 µg/mL aqueous solution of TPGS or a 500 µg/mL solution of TFD was separated in an Agilent InfinityLab Poroshell HPH-C18 column (2.1 × 100 mm, 1.9 µm) using a gradient elution method with two mobile phases. Mobile phase A comprised deionized water containing 10 mmol/L ammonium acetate and 0.1% formic acid, whereas mobile phase B was a mixture of methanol and isopropanol in a 2:3 ratio, also containing 10 mmol/L ammonium acetate and 0.1% formic acid. The flow rate of gradient elution was 0.4 mL/min under the following conditions: 0–3 min, 0%–100% mobile phase B; 3–10 min, 100% mobile phase B, with a2-minn re-equilibration using 0% mobile phase B. The injection volume was 1 µL, and the autosampler and column oven were maintained at 4℃ and 40℃, respectively. Electrospray ionization was configured to negative ionization mode for vitamin E analysis for the mass spectrometry settings. The parameters included a dry gas temperature of 200℃, a dry gas flow rate of 8 L/min, a nebulizer gas pressure of 2 bar, a capillary voltage of 4500 V, and an endplate offset potential of 500 V. Mass spectra were recorded within the range of 50 to 1500 m/z. The TOF mass analyzer was calibrated using sodium formate over a mass range of 50–1500 m/z for better accuracy. Each sample underwent duplicate analysis to ensure reliability and reproducibility (n = 2 technique

replicates). The LC-MS data revealed that the vitamin E peak exhibited identical retention times in both the TPGS and TFD solution peaks.

Results

Following the acid hydrolysis of the ester bond in TPGS and TFD, the resulting solutions were analyzed using LC–MS. TPGS, containing an ester bond, undergoes further hydrolysis to produce vitamin E. Quantification revealed that TPGS-FD contains a significant 25.7% (w/w) TPGS content (Figure S5A). Furthermore, we assessed the vitamin E content across various TFD concentrations, revealing a characteristic curve aligned with a mathematical equation. This typical linear regression equation is expressed as $y = mx + b$, where y signifies the measured signal (area), x represents the concentration of the analyzed TFD, m denotes the slope of the line, and b is the y-intercept. In our investigation, the specific linear regression equation employed was $y = 1956.7x + 221981.5$, accompanied by an R^2 value of 0.9999 (Figure S5B).

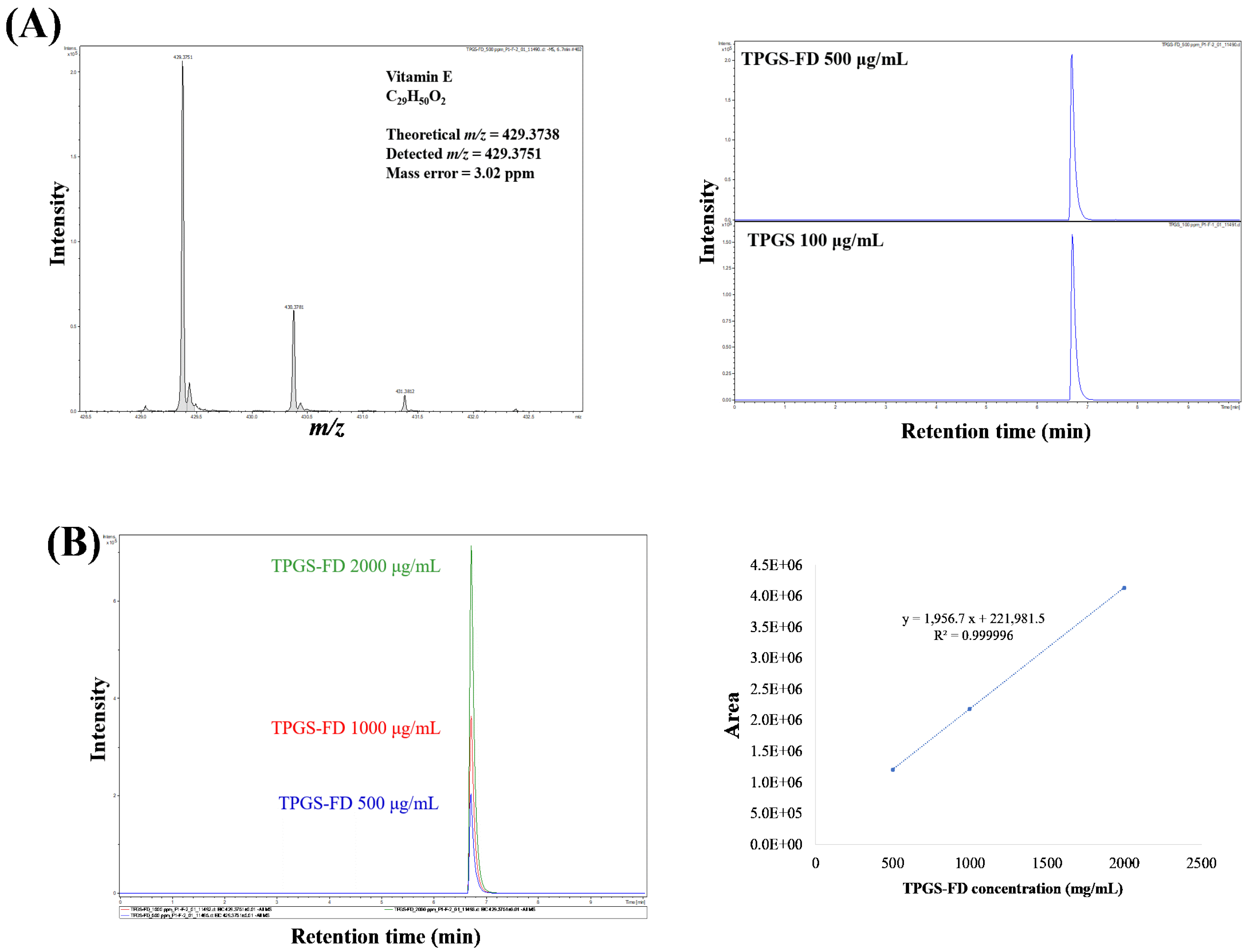

Figure S5. (A) Mass spectrum of vitamin E liberated from TPGS-FD, along with extracted ion chromatograms depicting released vitamin E from TPGS and TPGS-FD. (B) Extracted ion chromatograms and corresponding peak areas of vitamin E released from TPGS-FD at concentrations of 500, 1000, and 2000 μg/mL.

References

[1] Zhang, J.; Li, Y.; Fang, X.; Zhou, D.; Wang, Y.; Chen, M. TPGS-g-PLGA/Pluronic F68 Mixed Micelles for Tanshinone IIA Delivery in Cancer Therapy. *Int. J. Pharm.* **2014**, *476*. 185-198, DOI: 10.1016/j.ijpharm.2014.09.017.

[2] Chen, M. L.; Lai, C. J.; Lin, Y. N.; Huang, C. M.; Lin, Y. H. Multifunctional Nanoparticles for Targeting the Tumor Microenvironment to Improve Synergistic Drug Combinations and Cancer Treatment Effects. *J. Mater. Chem B.* **2020**, *8*, 10416-10427, DOI: 10.1039/d0tb01733g.

[3] Lei, H.; Ma, Q.; Wang, Z.; Zhang, D.; Huang, X.; Qin, M.; Ma, H.; Wang.; Cao, Y. Ester Bond: Chemically Labile yet Mechanically Stable. *ACS Nano* **2023**, *17*, 16870-16878. DOI: 10.1021/acsnano.3c03807.

[4] Bartella, L.; Mazzotti, F.; Napoli, A.; Sindona, G.; Di Donna, L. A Comprehensive Evaluation of Tyrosol and Hydroxytyrosol Derivatives in Extra Virgin Olive Oil by Microwave-Assisted Hydrolysis and HPLC-MS/MS. *Anal. Bioanal. Chem.* **2018**, *410*, 2193-2201. DOI: 10.1007/s00216-018-0885-1.

[5] Neophytou, C. M.; Constantinou, A. I. Drug Delivery Innovations for Enhancing the Anticancer Potential of Vitamin E Isoforms and Their Derivatives. *Biomed. Res. Int.* **2015**, *2015*, 584862. DOI: 10.1155/2015/584862.
